# Intrahepatic Cholestasis of Pregnancy Increases Inflammatory Susceptibility in Neonatal Offspring by Modulating Gut Microbiota

**DOI:** 10.3389/fimmu.2022.889646

**Published:** 2022-06-13

**Authors:** Qiong-xi Lin, Wan-wen Huang, Wei Shen, Xiao-shi Deng, Zi-yu Tang, Zhen-hui Chen, Wei Zhao, Hong-ying Fan

**Affiliations:** ^1^ Department of Microbiology, Guangdong Provincial Key Laboratory of Tropical Diseases Research, School of Public Health, Southern Medical University, Guangzhou, China; ^2^ Department of Neonatology, Nanfang Hospital, Southern Medical University, Guangzhou, China; ^3^ BSL-3 Laboratory (Guangdong), Guangdong Provincial Key Laboratory of Tropical Diseases Research, School of Public Health, Southern Medical University, Guangzhou, China

**Keywords:** intrahepatic cholestasis of pregnancy, farnesoid X receptor, gut microbiota, bile acids, inflammation, immune function

## Abstract

Intrahepatic cholestasis of pregnancy (ICP) is a liver disease of pregnancy that is characterized by increased bile acid levels in maternal serum. Studies have shown that cholestatic pregnancy can result in long-term metabolic disturbances in the offspring. However, how ICP shapes the offspring’s immunity and predisposition to inflammatory disorders at an early stage is unknown. In this study, we investigated the effect of maternal cholestasis on neonatal offspring metabolism and immune function. We compared 71 neonates with ICP mothers and 63 neonates with healthy mothers and found that the incidence of jaundice and infection was significantly higher in ICP offspring. Maternal serum total bile acid level was associated with blood cell counts in full-term ICP offspring. In animal experiments, a compensatory activation of hepatic and ileal farnesoid X receptor (FXR) and altered gut microbiota in the first week were found in ICP offspring. We also investigated lipopolysaccharide (LPS)-induced inflammatory responses in neonatal rats and found that ICP offspring were more susceptible to inflammation. To understand the correlation between congenital abnormal FXR activation and tissue immunity dysregulation, we assessed the effects of the FXR agonist GW4064 and FXR antagonist E/Z-GS in ICP offspring after LPS exposure. The expression of several pro-inflammatory cytokines significantly decreased after treatment with E/Z-GS but increased after treatment with GW4064. Treatment with the probiotic *Lactobacillus rhamnosus* LRX01 that inhibits FXR expression in the ileum reduced susceptibility to LPS exposure in ICP offspring. The current study indicated that cholestatic pregnancy may increase the susceptibility of the offspring to inflammation by altering bile acid metabolism and gut microbiota at an early stage. We suggest that supplementation with *Lactobacillus rhamnosus* LRX01, which inhibits FXR expression in the ileum, may improve intestinal immunity in ICP offspring.

## Introduction

Many adult diseases have been shown to originate *in utero* or during infancy (1, 2). The environment to which the mother is exposed can confer multiple congenital abnormalities and disease susceptibilities in the offspring (3). Intrahepatic cholestasis of pregnancy (ICP) is a liver disease occurring in pregnancy that is characterized by elevated levels of maternal serum bile acid (BA). Elevated BA levels in pregnant women are associated with adverse fetal outcomes in ICP, including spontaneous preterm labor, meconium-stained amniotic fluid, and fetal hypoxia (4). Studies have shown that cholestatic pregnancy can result in long-term metabolic disturbances in the offspring and elevate fetal BA levels in cord blood (5, 6). These studies indicate that abnormalities in hepatocytic BA may originate during the fetal period. However, how these disorders shape the health of the offspring at an early stage is unknown.

Bile acids are cholesterol-derived molecules that are synthesized in the liver and secreted into the duodenum. They are known to affect host metabolism and regulate enterohepatic circulation, mainly through the farnesoid X receptor (FXR) signaling pathways (7, 8). Recently, bile acids have been recognized as signaling molecules that regulate the host immune system by suppressing the NF-κB-dependent signaling pathway (9, 10), inhibiting NLRP3-dependent inflammasome activities (11), and modulating the Th17 and regulatory T cell balance (12, 13). BAs also affect intestinal associated inflammation, suggesting they have the potential to regulate intestinal mucosal immune cells. Therefore, we hypothesized that congenital abnormalities of BA metabolism may play a vital role in the immune imbalance in infants.

Fecal BA concentrations, immune cell abundance, and gut microbiota composition are closely related (14, 15). Evidence indicates that dysregulation of the immune system and cholesterol metabolism affects intestinal bacteria (16, 17). Bacteria and BAs also regulate immune tolerance in the human gut (18). The intestinal BAs and microbiome influence each other—bacteria modulate BA metabolism while intestinal BAs regulate the growth of symbiotic bacteria, maintain barrier integrity, and regulate the immune system (19). The co-occurrence of gut microbial dysbiosis and alterations in BA metabolism has been shown in pregnant women with ICP (20, 21), but the effects of this phenomenon on their offspring have not been investigated. Moreover, important features of cholestatic pregnancy, including factors that induce changes in the intestinal bacteria and immune system in the offspring, remain to be elucidated. An understanding of the molecular mechanisms underlying the regulation of the immune system by the biliary network between hosts and their associated microbiomes will be valuable in improving therapy for neonatal offspring in cholestatic pregnancy.

Collectively, we hypothesized that fetal exposure to higher intrauterine BA levels increases the susceptibility of the ICP offspring to inflammation at an early stage by modulating the gut microbial composition. Therefore, in this study, we investigated the effect of maternal cholestasis on neonatal offspring metabolism and immune function, as well as characterized the underlying mechanisms manifesting these effects.

## Methods

### Cohort Study

We conducted a single-center retrospective study approved by the Nanfang Hospital Institutional Review Board (NFEC-2021-430). All newborns assessed in this study were hospitalized between June 2014 and October 2021 at Nanfang Hospital (Guangzhou, China). 71 neonatal offspring of ICP mothers and 63 of normal mothers who did not have any other known maternal liver/metabolic disease were enrolled in the study. Demographic, clinical, and laboratory data of the participants, including clinical diagnosis, sex, gestational age, maternal total bile acid (TBA) before delivery, weight, liver function test index, and blood cell counts on the first day after birth, were collected from computerized medical records and the hospital laboratory database. All the clinical diagnoses adhered to the 10th edition of the International Classification of Diseases (ICD-10) criteria. For infection, all cases have one or more discharge diagnoses of infection (such as neonatal infectious pneumonia, neonatal syphilis, etc.), and were diagnosed by professional physicians based on the blood infection indexes (such as CRP, PCT, IL-6, etc.), clinical symptoms, or definite pathogen.

### Animal Handling

All rats were purchased from the Southern Medical University Experimental Animal Center and raised under specific pathogen-free conditions, with free access to commercial food and water before the experiments. All animal experiments were approved by the Ethics Committee of Southern Medical University (permit no. 44002100006397).

Sprague-Dawley (SD) rats aged between 9 to 12 weeks were used. The rats were housed in a temperature-and light-controlled room and mated overnight. A positive vaginal plug was designated embryonic day 0 of pregnancy. ICP was induced in the pregnant rat based on reported studies (5, 22). After plug identification, females were fed with a normal chow (NC) or 0.5% cholic acid (CA) diet from gestation day 16 until labor. The mothers were then switched to an NC diet after delivery. Half of the mothers and newborns were euthanized and sacrificed on the first day, and the other newborns were breastfed for seven days until sacrificed.

For the colitis induction experiment, newborn SD rats were breastfed for one day after birth and were hand-fed 60 µl of formula *via* an oral feeding tube (Abbott, USA) every 3h and administered lipopolysaccharide (LPS) (Solarbio, China, 10 mg/kg) for two days using oral gavage. The rats were sacrificed on the third day for further analysis.

For the FXR activating or inhibiting experiment, the newborn rats treated with the FXR agonist were administered GW4064 (50 mg/kg, Selleck, China) daily using an oral gavage for two days after birth. Rats treated with the FXR inhibitor were administered guggulsterone E-Z (10 mg/kg, Selleck, China) daily using an oral gavage for two days after birth. In the probiotic-treated group, rats received *Lactobacillus rhamnosus* LRX01(2 × 10^8^ CFU, 10ul) orally daily for the first two days after birth. *Lactobacillus rhamnosus* LRX01 was isolated from a healthy infant.

### Sample Collection

Animals were sacrificed by intraperitoneal pentobarbital (50 mg per kg body weight), perfused with 10-20ml phosphate-buffered saline (PBS) into the heart. After dissection, blood samples were centrifuged at 3,000 rpm for 15 min at -4°C, and then the sera were collected and stored at -80°C for further analysis. Liver, colon, spleen, and ileum tissue were stored at -80°C for further analysis. The stool samples were frozen in liquid nitrogen immediately and stored at -80°C for 16S rRNA gene sequencing and transcriptome analysis. Tissue samples from the liver, colon, and terminal ileum were taken for histological and immunohistochemical examinations and stored in 10% (v/v) formalin. The spleen index was calculated on the first postnatal day using the following formula: spleen index (mg/g) = spleen weight (mg)/animal body weight (g).

### Histology and Immunohistochemistry

The tissues were soaked and fixed with 10% formalin, embedded in paraffin, sectioned, and stained with hematoxylin and eosin (HE). For immunohistochemical staining, 5-mm-thick paraffin-embedded sections were deparaffinized and antigen retrieval was performed. The sections were blocked in 1% normal goat serum and incubated overnight at 4 °C with antibodies specific for FXR (Abcam, Cambridge, United Kingdom), then incubated with horseradish peroxidase (HRP)-coupled secondary antibodies in 50 mM Tris-HCl buffer (pH7.4) containing DAB (3,3-diaminobenzidine) and H_2_O_2_. A digital microscope camera (Nikon, Tokyo, Japan) was used to photograph the sections. Immunohistochemistry sample images were quantified using Image-Pro Plus software (Media Cybernetics, MD, USA).

### mRNA Extraction and Quantitative Real-Time PCR

Total RNA was extracted using the TRIzol reagent (Invitrogen, USA) according to the manufacturer’s instructions. The mRNA was then reverse transcribed into cDNA using reverse transcriptases (TaKaRa Bio, Japan). Real-time PCR was performed using a Roche LightCycler 96 Real-time System (Roche, Swiss). Each sample was analyzed in triplicates. The amplicons were quantified using SYBR Green fluorescence. The comparative threshold cycle (CT) method was used to quantify mRNA. Gene expression was determined using the CT values. All values were normalized to that of the housekeeping GAPDH gene. The primers used are listed in **Supplementary Table 1**.

### 16S rRNA Sequencing of Microbial DNA and Microbial Community Analysis

The offspring’s cecal content was analyzed on the seventh postnatal day. 16S rRNA amplicon sequencing and 16S rDNA gene analysis were performed by Meige Biotechnology Co., Ltd. Total genomic DNA from the cecal content was extracted using a bacterial DNA extraction kit (TaKaRa Bio, Japan) according to the manufacturer’s protocol. DNA concentration and purity were assessed using 1% agarose gels. The DNA was diluted to 1 ng/µL with sterile water. The V3-V4 hypervariable regions of the bacterial 16S rRNA gene were amplified using the following primers: 338F 5’-ACTCCTACGGGAGGCAGCAG-3’ and 806R 5’-GGACTACHVGGGTWTCTAAT-3’ using a thermocycler PCR system (GeneAmp 9700, ABI, USA). Subsequently, the library was quantified, normalized, and pooled. MiSeq v3 reagent was used to load samples for MiSeq sequencing. The software package QIIME (V1.9.1) was used to perform 16S rRNA data analyses. Sequences were clustered into operational taxonomic units (OTUs) at a 97% similarity cutoff using UPARSE (23) (http://drive5.com/uparse/), and chimeric sequences were identified and removed using USEARCH (http://www.drive5.com/usearch/). A representative tag was assigned to the different identified bacterial taxa using the Ribosomal Database Project (RDP) Classifier against the GenBank database, using a confidence threshold of 0.7.

Diversity analyses were conducted using abundance data from QIIME. The alpha diversity of the gut microbiota was measured using the Shannon, Chao1, and Simpson indices. The overall differences in the microbial community structure were evaluated using principal coordinate analysis (PCoA) of the unweighted-unifrac distance. Differential abundance analysis was performed using Student’s t-test at the phylum, family, genus, and OTU levels between the groups. BugBase (24) (https://bugbase.cs.umn.edu/index.html) was used to predict the organism-level microbiome phenotypes.

### Bile Acid Composition Analysis

The composition of bile acid from the offspring’s feces was analyzed according to a previously reported method (25). Stock solutions of all bile acids were prepared by dissolving the compounds in molecular biology grade Dimethylsulfoxide (DMSO). These solutions were used to establish a standard curve. Cholic acid (CA), Chenodeoxycholic acid (CDCA), Lithocholic acid (LCA), Beta-muricholic acid(β-MCA), Ursodeoxycholic (UDCA), Hyocholic acid (HCA), Hyodeoxycholic acid (HDCA), Taurocholic acid (TCA), Taurodeoxycholic acid (TCDCA), Tauro-beta-muricholic acid (T-β-MCA), Glycohyodeoxycholic acid (GHDCA), Glycolic acid (GCA) and Glycine-β-muricholic Acid (G-β-MCA) (Sigma-Aldrich) was used as internal standards. HPLC-grade solvents were used to prepare and run UPLC-MS samples. All data were analyzed using Agilent Chem Station.

### Cell Culture

The human epithelial colorectal adenocarcinoma cell line CaCo-2 (ECACC, No.86010202) was grown at 37 °C under 5% CO_2_ in high-glucose Dulbecco’s modified Eagle’s medium (DMEM; Wako, Osaka, Japan) supplemented with 10% heat-inactivated fetal bovine serum (Invitrogen, USA) and 1% penicillin-streptomycin solution (×100) (Wako, Japan). After seeding in a 6-well plate for 14 days, the cells were washed twice with 10 ml PBS and treated with 5 × 10^8^ CFU *Lactobacillus rhamnosus* LRX01 for 24 h. The cells were harvested for further analysis.

### Western Blot Analysis

The ileum tissues were completely minced with lysis buffer on ice. After the lysate was centrifuged at 4°C and 12,000 g for 10 min, the supernatant was collected. Nuclear protein extraction was performed by the instructions provided with the Nuclear and Cytoplasmic Protein Extraction Kit (KeyGen Biotech, China), and the protein concentration was determined using the BCA method. Extracts containing equal quantities of proteins (25 µg) were electrophoresed in 8 or 12% polyacrylamide gel. Subsequently, the separated proteins were transferred to a PVDF membrane. The membrane was blocked with 5% skimmed milk powder in TBS-Tween 20 buffer for 1 h and then incubated with a rabbit anti-IL-1β monoclonal antibody (1:1000 dilution; Proteintech, USA) overnight at 4°C. The membrane was subsequently incubated with secondary antibody (1:5000; Dingguo Biotechnology, China) for 1h at room temperature. Blots were developed using ECL detection reagents (Bio-Rad, USA).

### Cytokine Measurements

Rat serum was collected to determine the levels of IL-1β or TNF-α using ELISA kits (JSBOSSEN, China). The assay was performed in triplicate for each specimen, and the data was converted to pg/ml.

### Statistical Analysis

The difference between groups was analyzed using the chi-square test or Fisher’s exact test, Wilcoxon’s rank-sum test, Student’s t-test, or one-way analysis of variance. Pearson correlation analyses were performed to analyze the correlation between maternal TBA and immune indices of the offspring. Binary logistic regression was conducted to explore the association between infectious offspring and complications. Statistical analyses were performed using IBM SPSS 22.0 (IBM SPSS, USA), with significance set at p<0.05 (*p<0.05, **p<0.01, ***p<0.001). All plots were generated using GraphPad Prism 6.0 (GraphPad Software, San Diego, USA) and R packages (3.5.0).

## Results

### Cholestatic Pregnancy Is Associated With Infection in the Offspring

To explore the influence of a cholestatic pregnancy on offspring, we performed a retrospective investigation in 71 newborns of ICP mothers and 63 newborns (controls) of normal mothers who did not have any other known maternal liver/metabolic disease. Compared to that of the controls, the incidence of jaundice, infection and fetal distress in neonatal offspring was remarkably higher in ICP offspring (**Table 1**). No significant differences were observed in the sex or ratio of preterm to low-birth-weight infants between the two groups. In order to exclude the effect of intrauterine distress on the occurrence of infectious diseases in ICP offspring, we excluded 10 neonates with intrauterine distress in the ICP group and compared them with the control group. The results showed that the incidence of infectious diseases(χ^2^ = 5.324,p=0.021) and jaundice(χ^2^ = 5.141,p=0.023) in the neonates of the ICP group remains higher than in controls. To further verify the influencing factors of offspring infection, we performed the binary logistic regression on all cases, which showed that the ICP mother is an independent factor that influences infections in the offspring (OR = 3.137, 95% CI 1.225-8.036, p=0.017) (**Supplementary Table 2**).

**Table 1:** Comparison of clinical outcomes of neonates between the groups.

	ICP offspringn (%)	Control offspringn (%)	χ^2^	p
**Male neonate**	47 (66.2)	36 (57.1)	1.161	0.281
**Clinical outcomes**				
** Preterm birth**	31 (43.7)	25 (39.7)	0.217	0.641
** Low birth weight infant**	23 (32.4)	17 (27.0)	0.467	0.495
** Neonatal jaundice**	60 (84.5)	42 (66.7)	5.846^*^	0.016
** Neonatal infection**	20 (28.2)	7 (11.1)	6.037^*^	0.014
** Fetal distress**	10 (14.1)	0 (0)	12.775^*^	<0.001

ICP offspring n = 71; control offspring n = 63. One asterisk (*) denotes the p-value is <0.05.

**Figure 1:**
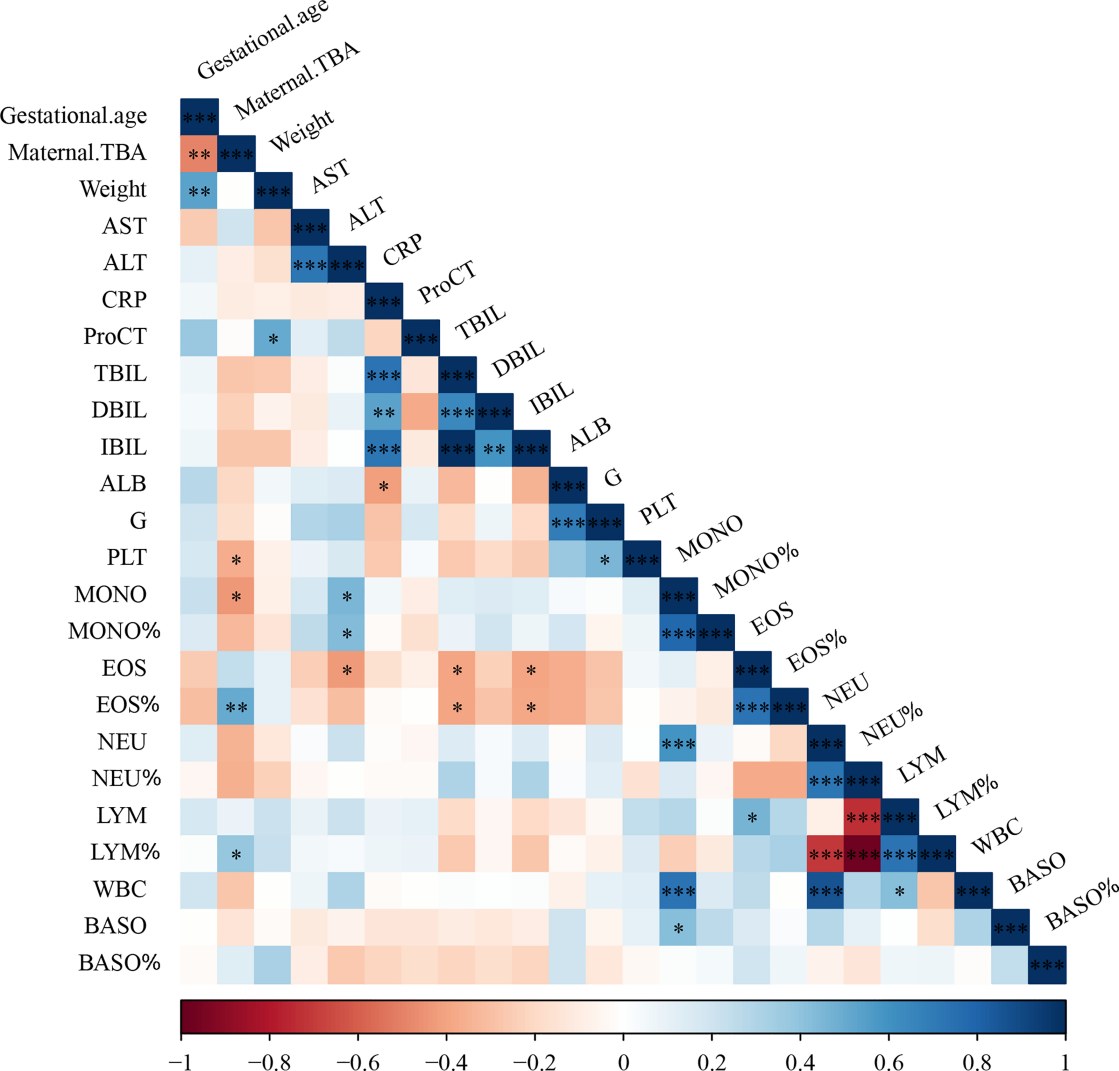
Cholestatic pregnancy is associated with hematologic parameters in the offspring. TBA, total bile acid; AST, aspartate aminotransferase; ALT, alanine transaminase; CRP, c-reactive protein; ProCT, procalcitonin; TBIL, total bilirubin; DBIL, direct bilirubin; IBIL, indirect bilirubin; ALB, albumin; G, globulin; PLT, Platelets; MONO, monocyte; EOS, eosinophil; NEU, neutrophil; LYM, lymphocyte; WBC, white blood cell; BASO, basophil; * p<0.05; ** p<0.01; *** p<0.001. N=29.

To further examine the relationship between maternal cholestasis and clinical parameters of the newborns, we analyzed the correlation between maternal serum TBA and blood cell counts of full-term neonatal offspring. As shown in **Figure 1**, maternal TBA correlated negatively with platelet (r=-0.368, p=0.049) and monocyte (r=-0.437, p=0.02) counts, whereas it correlated positively with the percentage of eosinophils (r=0.507, p=0.006) and lymphocytes (r=0.386, p=0.042). A trend of negative correlations between maternal TBA and neutrophil count (r=-0.347, p=0.065), and between maternal TBA percentage of neutrophils (r=-0.357, p=0.067) was also found. Besides, gestational age was negatively correlated with maternal TBA (r=-0.497, p=0.006) and positively correlated with offspring weight (r=0.537, p=0.003). C-reactive protein was positively correlated with total bilirubin (r=0.732, p<0.001), direct bilirubin (r=0.538, p=0.004), and indirect bilirubin (r=0.724, p<0.001) but negatively correlated with albumin (r=-0.418, p=0.027) in ICP offspring. These data suggest that ICP offspring may have a higher incidence of infectious diseases during the first week after birth, and various hematologic changes may be related to maternal TBA.

### Maternal Cholestasis During Pregnancy Affects Adverse Fetal Outcomes in Mammals

**Figure 2:**
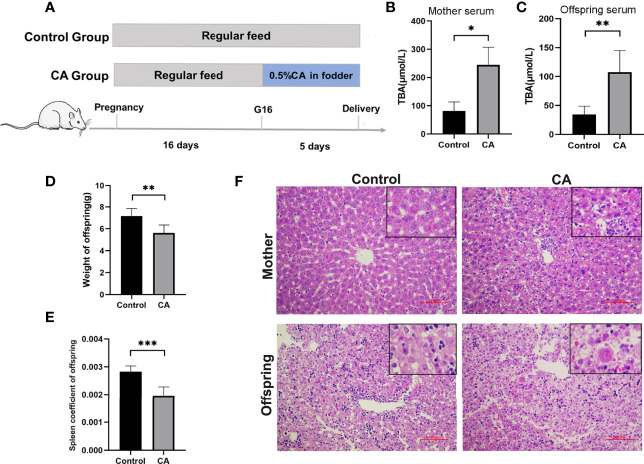
Maternal cholestasis during pregnancy affects adverse fetal outcomes in mammals. **(A)** Flow diagram of the experimental procedure. **(B)** Comparison of total bile acid in mother serum between two groups. **(C)** Comparison of total bile acid in fetal serum between two groups. **(D)** Comparison of offspring weight between two groups. **(E)** Comparison of offspring spleen index between two groups. **(F)** Microscopic observation of the ileum tissue with hematoxylin and eosin **(HE)** staining. Scale bar, 100 mm. Magnification, ×200 and ×400(insets). *p < 0.05, **p < 0.01, ***p<0.001. N = 3-8.

Furthermore, we developed a rat model of cholestatic pregnancy to investigate the subsequent outcomes in the offspring. Twelve SD female rats were randomly allocated to two groups with 0 or 0.5% (w/w) CA supplement diet at gestation day 16 and continued until labor. As shown in **Figure 2**, CA supplementation significantly increased maternal TBA, while no significant differences in AST and ALT levels were observed between the two groups. Accordingly, deformation and vacuolar degeneration of hepatocytes were observed in maternal livers consistent with a cholestatic phenotype. Compared to that of the control offspring, TBA in the serum increased and weight remarkably decreased in the offspring from CA-fed mothers. It is worth noting that increasing vacuolization was also found in the offspring from CA-fed mothers, indicating a congenital abnormality of liver function. In addition, we found that the spleen index in the offspring from CA-fed mothers was significantly lower than that in the controls.

### Enterohepatic Circulation of Bile Acid Is Dysregulated in the Offspring From CA-Fed Mothers

**Figure 3:**
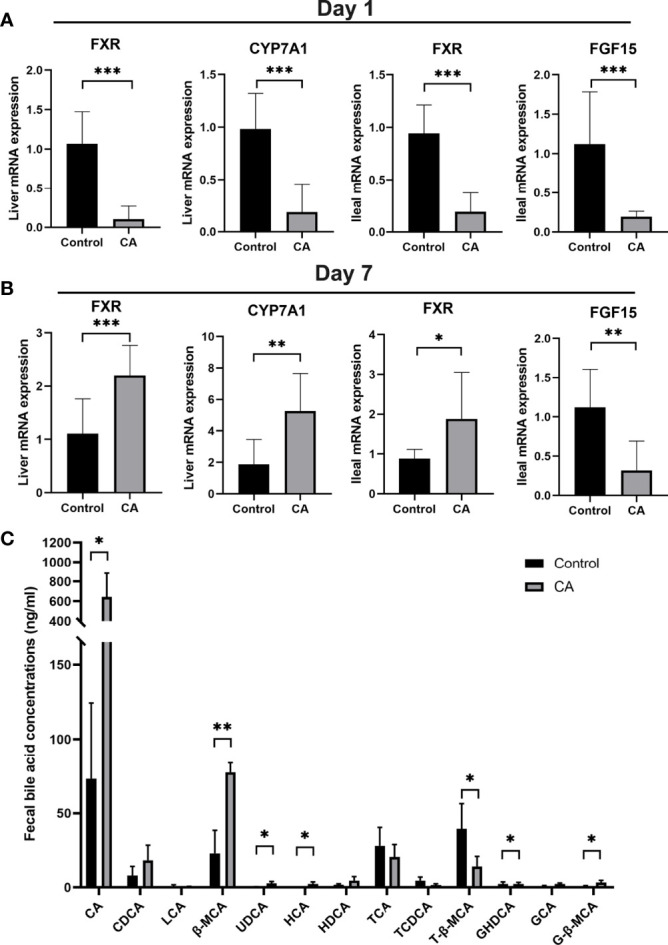
Enterohepatic circulation of bile acid is dysregulated in the offspring from CA-fed mothers. **(A)** Relative mRNA expression levels of FXR, CYP7A1 in liver and FXR, FGF15 in ileum of first day offspring from CA-fed mothers. **(B)** Relative mRNA expression levels of FXR, CYP7A1 in liver and FXR, FGF15 in ileum of seven days offspring from CA-fed mothers. **(C)** Major bile acids found in feces. *p < 0.05, **p < 0.01, ***p<0.001. N = 3-6.

To explore the effects of maternal cholestasis on the enterohepatic circulation of BA in the offspring from CA-fed mothers, genes related to bile acid metabolism in the liver and ileum were assayed. On postnatal day 1, hepatic FXR, CYP7A1, ileal FXR, and FGF15 mRNA were significantly reduced in the offspring from CA-fed mothers than in the controls (**Figure 3A**). However, on the 7th postnatal day, hepatic FXR, CYP7A1, and ileal FXR mRNA were markedly increased but ileal FGF15 mRNA was still notably reduced (**Figure 3B**).

In addition, targeted UPLC-MS/MS evaluation feces of offspring to analyze bile acid metabolism revealed lower conjugated bile acids in the offspring from CA-fed mothers, with proportionately less T-β-MCA, TCA, and more CA, β-MCA, UDCA, HCA, GHDCA and G-β-MCA (**Figure 3C**). These results suggest that congenital disorders of the enterohepatic circulation in ICP offspring can lead to abnormal activation of hepatic and ileum FXR and impair bile acid metabolism in the early stage.

### Gut Microbiota Is Altered in the Offspring From CA-Fed Mothers

**Figure 4:**
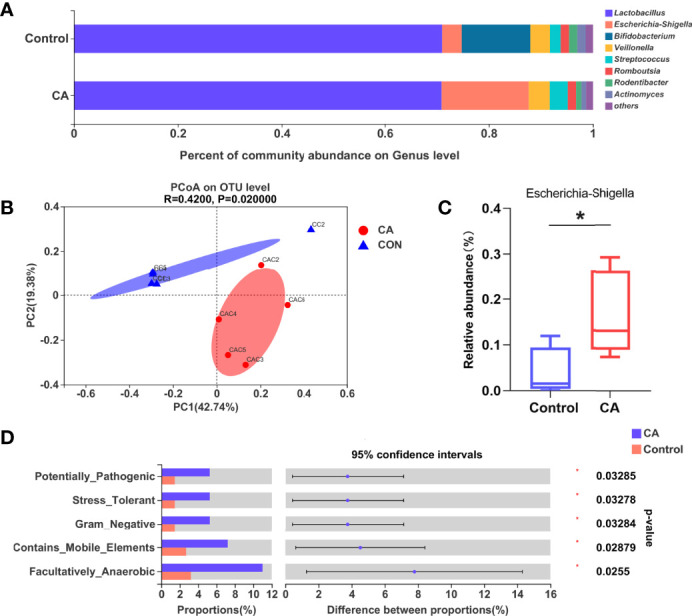
Gut microbiota is altered in the offspring from CA-fed mothers. **(A)** Composition of gut microbiota of two groups on genus level. **(B)** PCoA on OTU level. **(C)** Relative abundance of *Escherichia Shigella* genera in two groups. **(D)** Microbial phenotype prediction by BugBase. *p < 0.05. N = 4-5.

As the gut microbiota plays an important role in FXR expression and bile acid transfer, we analyzed the offspring’s gut microbiota at postnatal day 7. The nine most dominant microbial genera identified in the cecal contents are shown in **Figure 4A**. A remarkable difference in the β-diversity of the gut microbiota was found between offspring of the two groups suggesting that maternal cholestasis affected the gut microbial diversity of offspring (**Figure 4B**). No differences were detected in the α-diversity indices. Comparison between groups shows that the relative abundance of *Escherichia Shigella* was higher in the offspring from CA-fed mothers than in the controls. To further understand how this difference in microbial composition manifests, we used BugBase to predict the bacterial characteristics. We identified that gene functions associated with the potentially pathogenic, gram-negative, stress-tolerant, mobile element-containing, and facultative anaerobic phenotypes were enriched in the offspring from CA-fed mothers. These results indicate that *Escherichia Shigella* (**Figure 4C**) and gram-negative bacteria prediction (**Figure 4D**) were more abundant in the offspring from CA-fed mothers, which may cause an increase in LPS levels and increase the risk of inflammatory infection.

### Offspring From CA-Fed Mothers Showed a Predisposition to Inflammatory Disorders

**Figure 5:**
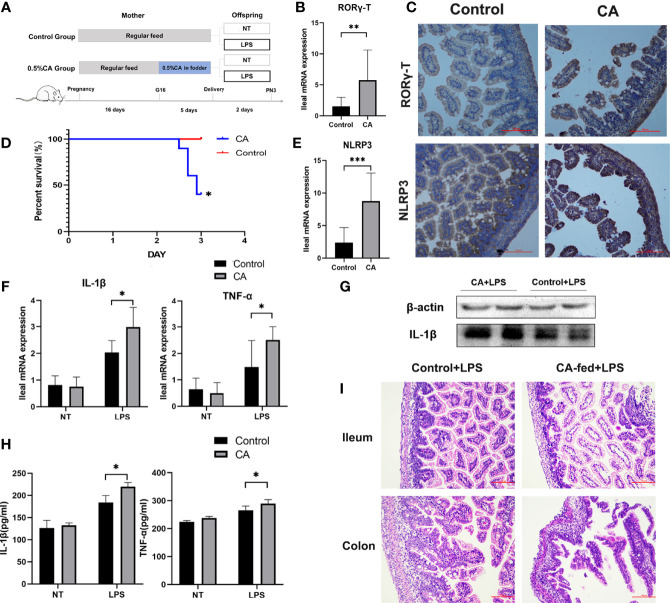
Offspring from CA-fed mothers showed a predisposition to inflammatory disorders. **(A)** Flow diagram of the experimental procedure. **(B, E)** Ileum mRNA expression of RORγ-T and NLRP3 in two groups. **(C)** Immunohistochemical staining for RORγ-T and NLRP3 (brown) in the 1st day ileum tissue section. **(D)** Survival analyses after LPS exposure. **(F)** Ileal mRNA expression of IL-1β and TNF-α upon LPS exposure in two groups. **(G)** Western blot analysis of IL-1β in the ileum. **(H)** IL-1β and TNF-α serum levels upon LPS exposure in two groups. **(I)** Microscopic observation of the ileum and colon tissue with hematoxylin and eosin (HE) staining. Scale bar, 100 mm. Magnification, ×200. *p < 0.05, **p < 0.01, ***p<0.001. N =3-6.

As FXR has been reported to regulate the activation of the NLRP3 inflammasome and T cell function (13, 26), we measured the expression of *Foxp3*, *T-bet*, GATA-3, RORγ-T, and NLRP3 as immunity and inflammation intermediate factors on postnatal day 1 in offspring. As shown in **Figures 5B, C, E**, a significantly increased mRNA expression of RORγ-T and NLRP3 was found in the offspring from CA-fed mothers, which was also verified at the protein level. However, there were no significant differences in Foxp3, T-bet, and GATA-3 levels between the two groups (**Supplementary Figure 1A**).

To further observe whether the offspring from CA-fed mothers are susceptible to inflammatory disorders, we investigated LPS-induced inflammatory responses in neonatal rats (**Figure 5A**). We found that after two days of LPS treatment, the mortality rate was significantly higher in the offspring from CA-fed mothers than in the controls (**Figure 5D**). We also observed a considerable increase in mRNA expression and serum levels of pro-inflammatory cytokines IL-1β and TNF-α (**Figures 5F–H**). RORγ-T and NLRP3 expression levels were also activated in the offspring from CA-fed mothers after LPS exposure (**Supplementary Figure 1B**). In addition, histological analysis revealed that morphological alterations and acute epithelial degeneration in the ileum and colon after LPS exposure were more prominent in the offspring from CA-fed mothers than in the controls (**Figure 5I**). This data is consistent with the inflammatory responses observed on postnatal day 1, indicating that offspring from CA-fed mothers are more susceptible to developing inflammatory disorders than normal offspring.

### FXR Mediates Intestinal Immune Responses to LPS in the Offspring From CA-Fed Mothers

**Figure 6:**
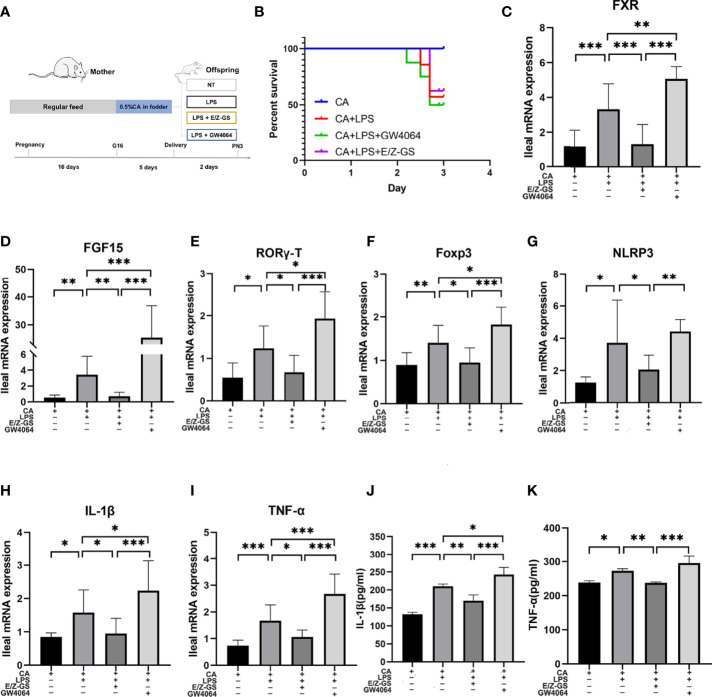
FXR mediates intestinal immune responses to LPS in the offspring from CA-fed mothers. **(A)** Flow diagram of the experimental procedure. **(B)** Survival analyses of four groups. **(C–I)** Ileal mRNA expression of FXR, FGF15, IL-1β, TNF-α, RORγ-T, Foxp3 and NLRP3. *p < 0.05, **p < 0.01, ***p<0.001. N = 5-7. **(J, K)** IL-1β and TNF-α serum levels in four groups.

To further validate the role of FXR in inflammation, we used an agonist (GW4064) and antagonist (E/Z-GS) of FXR to treat offspring from CA-fed mothers before LPS exposure (**Figure 6A**). As shown in **Figure 6B**, treatment with the FXR antagonist E/Z-GS reduced the mortality rate and RORγ-T, Foxp3, and NLRP3 expression by inhibiting the transcription of FXR and FGF15 in the ileum (**Figures 6C, D**). The ileal mRNA expression and serum levels of TNF-α and IL-1β were also decreased in the E/Z-GS group (**Figures 6H–K**). Conversely, it was found that treatment with FXR agonist GW4064 caused the highest mortality rate among the four groups and significantly activated the proinflammatory cytokines IL-1β and TNF-α. By activating the transcription of FXR and FGF15 in the ileum, the expressions of NLRP3, RORγ-T, and Foxp3 in ileum were also increased in the GW4064 group (**Figures 6E–G**). These results confirm that FXR mediates the intestinal immune response of offspring from CA-fed mothers to an immune challenge, such as *via* LPS exposure.

### Bacteria-Mediated Inhibition of FXR Reduced Susceptibility to LPS Exposure in the Offspring From CA-Fed Mothers

**Figure 7:**
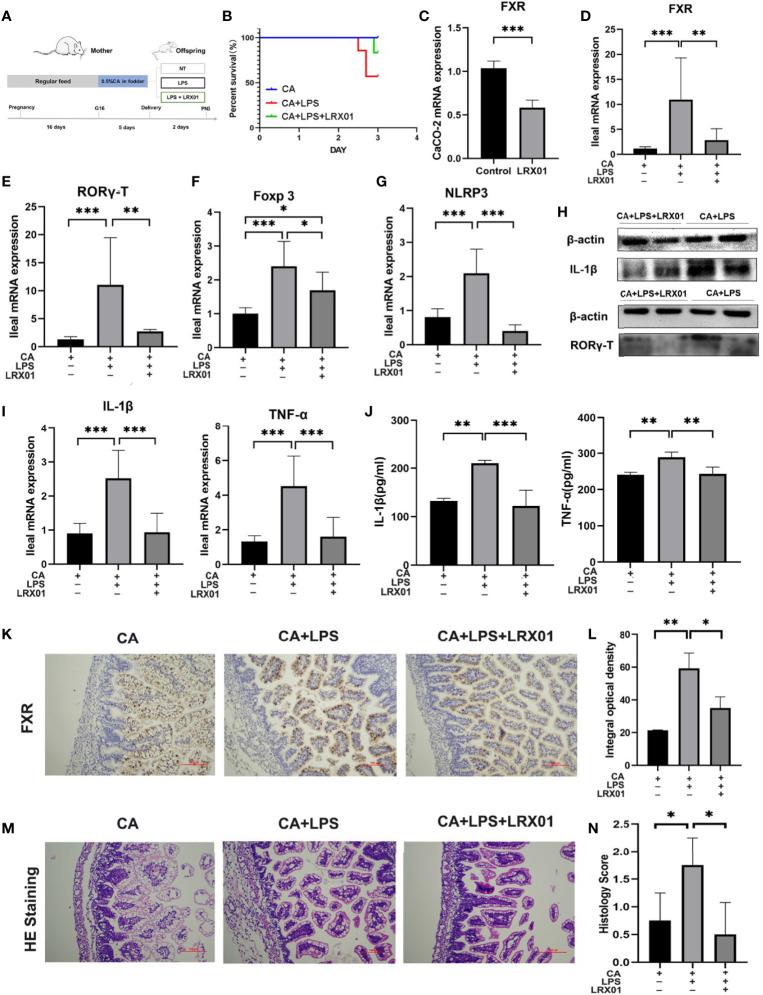
Bacteria-mediated inhibition of FXR reduced susceptibility to LPS exposure in the offspring from CA-fed mothers. **(A)** Flow diagram of the experimental procedure. **(B)** Survival analyses of three groups. **(C)** FXR mRNA expression in CaCO-2 experiment. **(D–G)** Ileal mRNA expression of FXR, RORγ-T, Foxp3, and NLRP3 in rats. **(H)** Western blot analysis of IL-1β and RORγ-T in ileum. **(I)** Ileal mRNA expression of IL-1β and TNF-α. **(J)** IL-1β and TNF-α serum levels in three groups **(K)** Immunohistochemical staining for FXR (brown) in ileum tissue section. **(L)** Comparison of integral optical density among groups. **(M)** Microscopic observation of the ileum tissue with hematoxylin and eosin (HE) staining. **(N)** Comparison of histology scores among groups. Scale bar, 100 mm. Magnification, ×200. *p < 0.05, **p < 0.01, ***p<0.001. N =3-4.

**Figure 8:**
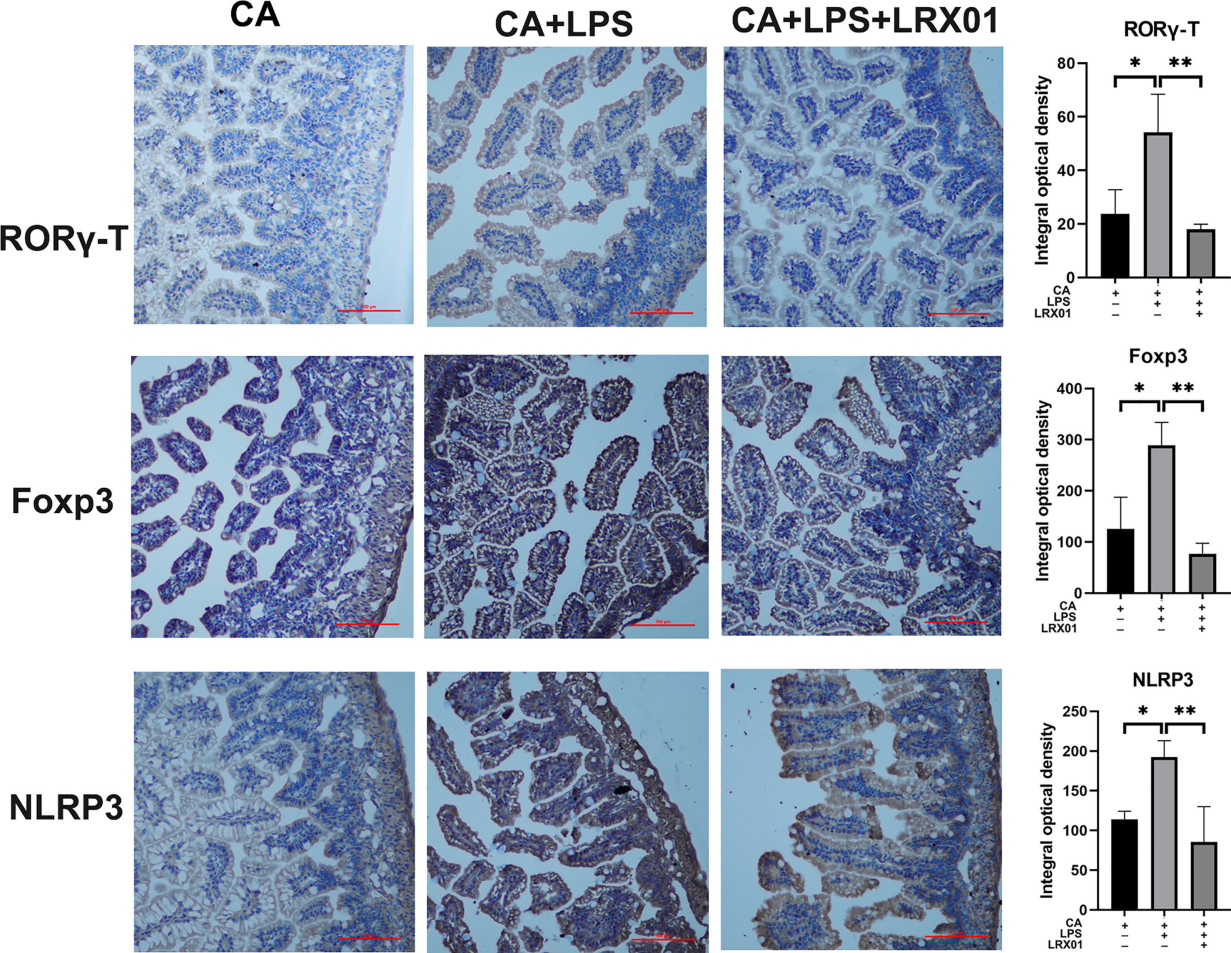
Immunohistochemical staining for RORγ-T, Foxp3, and NLRP3 of ileum treated with LRX01. *p < 0.05, **p < 0.01.

To investigate whether bacteria-mediated inhibition of FXR could improve intestinal immune responses, we treated CA-fed mothers’ offspring with *Lactobacillus rhamnosus* LRX01 before LPS exposure (**Figure 7A**), which has been shown to significantly reduce the expression of FXR in Caco-2 cells (**Figure 7C**) and the ileum (**Figures 7D, K, L**). As shown in **Figure 7B**, treatment with LRX01 remarkably reduced the mortality rate and the expression of RORγ-T, Foxp3 and NLRP3 in the ileum (**Figures 7E–G**, **Figure 8**). In addition, the ileal mRNA expression and serum levels of IL-1β and TNF-α in the offspring from CA-fed mothers were also improved after LRX01 treatment (**Figures 7I, J**), the protein levels of IL-1β were also verified (**Figure 7H**). Histological analysis revealed a marked improvement in epithelial degeneration and decreased histological score in the LRX01-treated offspring (**Figures 7M, N**). These results indicate that LRX01 supplementation has the potential to reduce susceptibility to LPS exposure in the offspring from CA-fed mothers by inhibiting ileal FXR expression.

## Discussion

**Figure 9:**
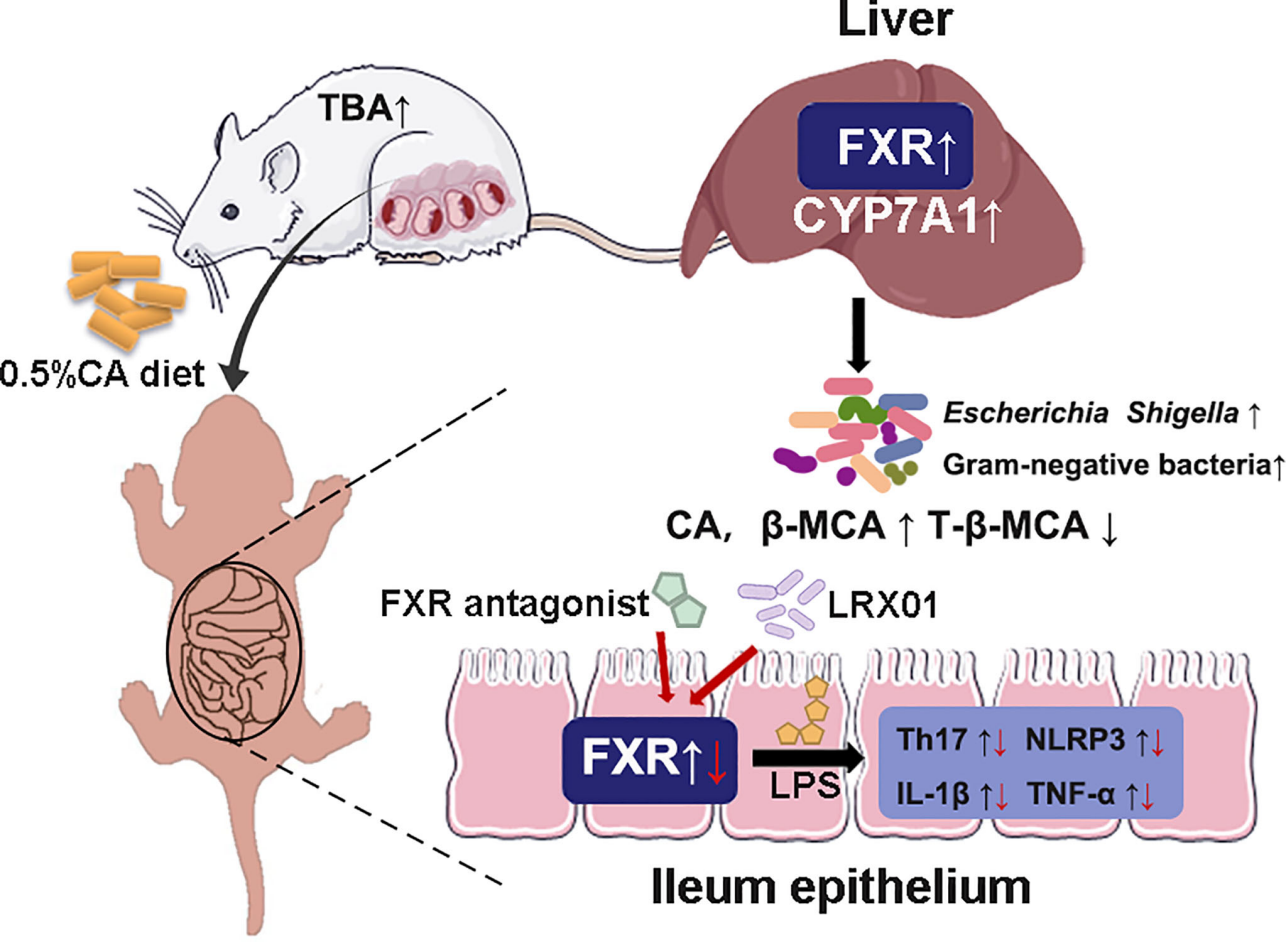
Schematic illustration of the role of FXR in the regulation of inflammation in ICP offspring.

Intrahepatic cholestasis of pregnancy (ICP) is a dangerous pregnancy complication that can lead to adverse outcomes in the offspring. However, few studies have investigated how ICP shapes offspring health at an early stage of life. In this study, we demonstratedthat fetal exposure to elevated BA levels *in utero* in ICP increased the susceptibility of the offspring to inflammation by altering enterohepatic circulation and gut microbiota at an early stage. In addition, we showed that supplementation with *Lactobacillus rhamnosus* LRX01, which inhibits FXR expression in the ileum, has the potential to reduce susceptibility to LPS exposure in the offspring from CA-fed mothers (**Figure 9**).

Accumulating evidence suggests that intrahepatic cholestasis during pregnancy has a substantial influence on both maternal and fetal health (27, 28). A previous study revealed that elevated maternal BA in pregnant swine leads to dysregulated BA metabolism in the fetus (29). Another study also showed that cholestatic pregnancy could program long-term metabolic disturbances in the offspring (5). These studies suggest that offspring of mothers with elevated BA may suffer from abnormalities in hepatocytic bile acid from the fetal period. We also found that among hospitalized newborns, the incidence of jaundice, fetal distress and infection in newborns with an ICP mother was significantly higher than that in newborns with a healthy mother. In addition, we found that maternal serum TBA was associated with blood cells in ICP offspring. A few studies have reported on infectious diseases in the early stages of ICP offspring. Recently, a study reported that cytomegalovirus infection was considerably more frequent in preterm infants with cholestasis than in non-cholestatic offspring, and more necrotizing enterocolitis was prevalent among cytomegalovirus-positive cholestatic infants (30). Combined with our results, we postulate that ICP offspring may be more susceptible to inflammatory diseases.

Some studies have reported that bacteria-regulated hepatocytic bile acid plays a vital role in immune imbalance and intestinal health (12, 31, 32), and liver complications are commonly observed in patients with inflammatory bowel diseases (33). These studies have suggested that cholestatic pregnancy may affect metabolic disorders and predispose neonatal offspring to inflammation. Animal experiments have also shown that maternal cholestasis in pregnancy leads to adverse outcomes and metabolic disturbances in neonatal rats. The current study found that the hepatic and ileal FXR mRNA were significantly inhibited on day 1 and activated on day 7 in the CA group, suggesting that there is a compensatory activation of hepatic and ileal FXR occurring during the first week of life. We also analyzed the bile acid composition of feces and gut microbiota of the one-week offspring, all of which showed abnormal performance in the CA group. These results showed that offspring from CA-fed had abnormal enterohepatic circulation in the first week. Emerging evidence suggests that BA metabolites regulate host immune responses by directly modulating the NLRP3 inflammasome and T cell balance (13, 26). Our results showed increased expression of RORγ-T and NLRP3 in the offspring from CA-fed mothers during the first week of birth. These results indicate that congenital abnormalities of BA metabolism in the offspring from CA-fed mothers may lead to disturbed immune homeostasis and inflammatory susceptibility. As more gram-negative bacteria were observed in gut bacteria of the offspring from CA-fed mothers, we investigated LPS-induced inflammatory responses in neonatal rats and found increased pro-inflammatory cytokines including IL-1β and TNF-α, and the expression of NLRP3/Th17 cells (RORγ-T). Th17 cells produce many pro-inflammatory cytokines, such as IL17 and TNF-α, that play critical roles in inflammatory disease and immune homeostasis (34, 35). A previous study showed that activation of the NLRP3 inflammasome induces caspase-1 activation and the production of the inflammatory cytokine IL-1β (26), which is consistent with the current study. To further confirm the role of FXR in immune dysregulation, we used FXR agonist GW4064 and antagonist E/Z-GS treatment in the offspring from CA-fed mothers before LPS exposure. As expected, the antagonist of FXR enhanced the intestinal immune responses of offspring from CA-fed mothers to LPS, suggesting that ileal FXR may be targeted for improving early intestinal immune disorders in the offspring from CA-fed mothers.

We also found an increased abundance of *Escherichia Shigella* and other gram-negative bacteria in ICP offspring. These observations are consistent with that of a study that showed that *Escherichia Shigella* is enriched in severe ICP patients (36), indicating that the gut of cholestatic mothers and fetuses harbor pathogenic bacteria. Targeted UPLC-MS/MS evaluation of bile acids in feces showed that T-β-MCA, an antagonist of FXR (37), was found to be reduced in ICP offspring. Studies have shown that gut microbiota plays an important role in FXR expression and bile acid metabolism (38). Therefore, we first used *Lactobacillus Rhamnosus* LRX01 to inhibit FXR expression in the ileum to assess if it reduces susceptibility to LPS exposure in the offspring from CA-fed mothers. A recent study showed that *Lactobacillus Rhamnosus* GG (LGG) treatment significantly decreased liver and ileum inflammation in bile duct ligation mice, suggesting that LGG may improve inflammation in BDL mice by increasing FXR activity (39). However, we found that the inhibition of the FXR improved intestinal inflammation in the offspring from CA-fed mothers. According to previous studies (40), we believe that this discrepancy is mainly due to the differences in the disease models between the two studies. In our study, the abnormal activation of ileal FXR was observed in the offspring from CA-fed mothers, suggesting that FXR inhibitors play a protective role in the offspring from CA-fed mothers’ immune function.

The current study has several limitations. First, we confirmed that LRX01 improved intestinal immunity of offspring from CA-fed mothers by negatively regulating FXR, however, whether LRX01 regulates the immune system through other pathways is unknown. In addition, our study only focused on the immunity of ICP offspring in the early stage, and the development of immunity in the later stages needs to be investigated in future studies. Moreover, studies (5, 41) have suggested that maternal cholestasis affects lipid biosynthesis and bile acid transport in the placenta, its relationship with the early fetal immune response remains to be further elucidated. In the cohort study, given that all the neonates were hospitalized and suffered from various diseases, which might affect the blood cell counts directly, we did not compare the blood cell counts of neonates between the two groups. A prospective cohort study may be considered to further explore this issue in the future. Despite these shortcomings, the current study demonstrates the potential to use the gut microbiome to improve the inflammatory susceptibility in the offspring from cholestatic mothers. However, this study is only a preliminary exploration of bacteria-mediated inhibition of FXR to improve intestinal immune responses and many issues are yet to be elucidated.

In conclusion, our results showed that maternal cholestasis increases the susceptibility of the offspring to inflammation by altering bile acid metabolism and gut microbiota at an early stage. We found that by modulating the microbiota, such as supplementation with *Lactobacillus rhamnosus* LRX01, the gut immunity of ICP offspring was improved by suppressing FXR expression in the ileum.

## Data Availability Statement

The original contributions presented in the study are publicly available. This data can be found here: https://www.ncbi.nlm.nih.gov/bioproject/PRJNA812501.

## Ethics Statement

The studies involving human participants were reviewed and approved by Nanfang Hospital Institutional Review Board (NFEC-2021-430). Written informed consent from the participants’ legal guardian/next of kin was not required to participate in this study in accordance with the national legislation and the institutional requirements. The animal study was reviewed and approved by Ethics Committee of Southern Medical University (permit no. 44002100006397).

## Author Contributions

H-YF and Q-XL: study design, statistical analysis, and manuscript preparation. W-WH and WS: data collection, animal and cell experiments operation. Z-YT and X-SD: literature search and microbiota analysis. Z-HC and WZ: data interpretation and instrument operation. All authors contributed to the article and approved the submitted version.

## Funding

This work was supported by National Natural Science Foundation of China (No. 32070118, 31872630), Guangdong Basic and Applied Basic Research Foundation (No. 2019A1515011759), Science and Technology Planning Project of Guangdong Province (No.2018B020205002, 2021B1212030014), Guangdong Medical Research Foundation (No. A2021191, B2022154) and Wu Jieping medical foundation (320.6750.2021-06-22).

## Conflict of Interest

The authors declare that the research was conducted in the absence of any commercial or financial relationships that could be construed as a potential conflict of interest.

## Publisher’s Note

All claims expressed in this article are solely those of the authors and do not necessarily represent those of their affiliated organizations, or those of the publisher, the editors and the reviewers. Any product that may be evaluated in this article, or claim that may be made by its manufacturer, is not guaranteed or endorsed by the publisher.
