# Supplementary material for: Intrahepatic Cholestasis of Pregnancy Increases Inflammatory Susceptibility in Neonatal Offspring by Modulating Gut Microbiota
Source: Front Immunol. 2022 Jun 13;13:889646. doi: 10.3389/fimmu.2022.889646 (PMC9234109; doi:10.3389/fimmu.2022.889646)
Supplement: Supplementary file 1 [file DataSheet_1.pdf]

Supplementary Table1: Primers for Real-time PCR

Gene	Primer orientation	Neucleotide sequence
GAPDH	forward	5'-AGCTTGTCATCAACGGGAAG-3'
	reverse	5'-TTTGATGTTAGTGGGGTCTCG-3'
FXR	forward	5'-TCGTAGAATTCACAAAAAGACTTCCAG-3'
	reverse	5'-AAGTTTCTTATTGAAAATCTCCGCTGAA-3'
FGF15	forward	5'-GGCTGATTCGCTACTCGGAGGA-3'
	reverse	5'-TGTGGAGGTGGTGCTTCATGGA-3'
CYP7A1	forward	5'-TGCATATGGTTTATTGTTGGAATAAGGA-3'
	reverse	5'-ATTAGCTCTTAGGAACTCAAGAGGATTA-3'
IL-1β	forward	5'-TACCTATGTCTTGCCCGTGGAG-3'
	reverse	5'-ATCATCCCACGAGTCACAGAGG-3'
TNF-α	forward	5'-AAATGGGCTCCCTCTCATCAGTTC-3'
	reverse	5'-TCTGCTTGGTGGTTTGCTACGAC-3'
Foxp3	forward	5'-ATGTTCGCCTACTTCAGA-3'
	reverse	5'-CCTTCTCACTCTCCACTC-3'
RORγ-T	forward	5'-GTCCAGAGATGCTGTCAA-3'
	reverse	5'-GGAGTCTTGGCTACTTGTT-3'
GATA3	forward	5'-CCACCCCATTACCACCTATCC-3'
	reverse	5'-CGGTTCTGCCCATTCATTTT-3'
T-bet	forward	5'-TTGGAAGGTGCCCGACTAAC-3'
	reverse	5'-AGGAAACAAGTGAACCCCAGAA-3'
NLRP3	forward	5'-AACTTGCAGAAGCTGGGGTT-3'
	reverse	5'-CAGAACCTCACAGAGCGTCA-3'

Supplementary Table 2: Binary logistic regression analysis of infections in offspring

Item	B	S.E	P	OR	95% CI
**ICP**	1.143	0.480	0.017	3.137	1.225-8.036
**Constant**	-2.079	0.401	<0.001	0.125	

B, partial regression coefficient; SE, Standard Error; CI, confidence interval; OR, odds ratio.

Supplementary Table 3 The type of infection in two groups.

Infection type	ICP(n)	Control(n)
Respiratory infection	1	2
GI infection	5	2
Peripheral infection	2	1
Other infection (no specific)	14	2

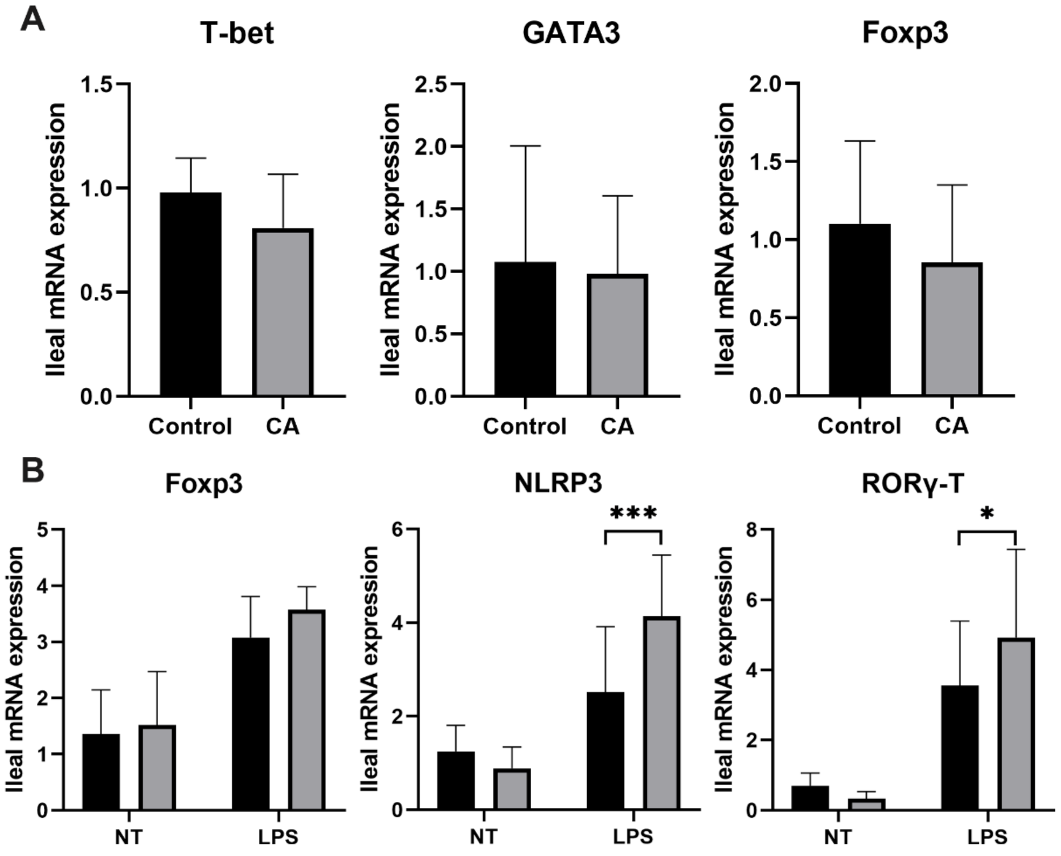

Supplementary Fig.1 The mRNA expression of ileal genes. (A)The mRNA expression of T-bet, GATA3 and Foxp3 on the 1th day of life. (B) The mRNA expression of Foxp3, NLRP3 and RORγ-T after LPS exposure. *$p < 0.05$, **$p < 0.01$, ***$p<0.001$. N =4-6.
